# A multicentric, prospective study on oral and maxillofacial trauma in the female population around the world

**DOI:** 10.1111/edt.12750

**Published:** 2022-04-07

**Authors:** Irene Romeo, Federica Sobrero, Fabio Roccia, Sean Dolan, Sean Laverick, Kirsten Carlaw, Peter Aquilina, Alessandro Bojino, Guglielmo Ramieri, Francesc Duran‐Valles, Coro Bescos, Ignasi Segura‐Pallerès, Dimitra Ganasouli, Stelios N. Zanakis, Luis Fernando de Oliveira Gorla, Valfrido Antonio Pereira‐Filho, Daniel Gallafassi, Faverani Leonardo Perez, Haider Alalawy, Mohammed Kamel, Sahand Samieirad, Mehul Raiesh Jaisani, Sajjad Abdur Rahman, Tabishur Rahman, Timothy Aladelusi, Ahmed Gaber Hassanein, Maximilian Goetzinger, Gian Battista Bottini

**Affiliations:** ^1^ Division of Maxillofacial Surgery Città della Salute e della Scienza University of Turin Turin Italy; ^2^ 3042 Department Oral and Maxillofacial Surgery University of Dundee Dundee UK; ^3^ Department Plastic, Reconstructive and Maxillofacial Surgery Nepean Hospital Sydney New South Wales Australia; ^4^ Department Oral and Maxillofacial Surgery Hospital Universitario Vall D’Hebron Barcelona Spain; ^5^ Department Oral and Maxillofacial Surgery Hippokration General Hospital Athens Greece; ^6^ Department Diagnosis and Surgery Araraquara Dental School São Paulo State University UNESP Araraquara, São Paulo Brazil; ^7^ Division of Oral and Maxillofacial Surgery Department of Diagnosis and Surgery São Paulo State University UNESP Araçatuba, São Paulo Brazil; ^8^ Department Oral and Maxillofacial Surgery Gazi Alhariri Hospital Medical City Baghdad Iraq; ^9^ Oral and Maxillofacial Diseases Research Center Mashhad University of Medical Sciences Mashhad Iran; ^10^ Department Oral and Maxillofacial Surgery Dharan Nepal; ^11^ 30037 Department Oral and Maxillofacial Surgery Aligarh Muslim University Aligarh India; ^12^ Department Oral and Maxillofacial Surgery College of Medicine University of Ibadan Ibadan Nigeria; ^13^ Maxillofacial Surgery Unit Faculty of Medicine General Surgery Department Sohag University Sohag Egypt; ^14^ Department Oral and Maxillofacial Surgery Paracelsus Medical University Salzburg Austria

**Keywords:** epidemiology, female, maxillofacial fractures, multicentric, prospective study

## Abstract

**Background/Aims:**

Approximately 20% of patients with maxillofacial trauma are women, but few articles have analysed this. The aim of this multicentric, prospective, epidemiological study was to analyse the characteristics of maxillofacial fractures in the female population managed in 14 maxillofacial surgery departments on five continents over a 1‐year period.

**Methods:**

The following data were collected: age (0–18, 19–64, or ≥65 years), cause and mechanism of the maxillofacial fracture, alcohol and/or drug abuse at the time of trauma, fracture site, Facial Injury Severity Scale score, associated injury, day of trauma, timing and type of treatment, and length of hospitalization.

**Results:**

Between 30 September 2019 and 4 October 2020, 562 of 2387 patients hospitalized with maxillofacial trauma were females (24%; M: F ratio, 3.2:1) aged between 1 and 96 years (median age, 37 years). Most fractures occurred in patients aged 20–39 years. The main causes were falls (43% [median age, 60.5 years]), which were more common in Australian, European and American units (*p* < .001). They were followed by road traffic accidents (35% [median age, 29.5 years]). Assaults (15% [median age, 31.5 years]) were statistically associated with alcohol and/or drug abuse (*p* < .001). Of all patients, 39% underwent open reduction and internal fixation, 36% did not receive surgical treatment, and 25% underwent closed reduction.

**Conclusion:**

Falls were the main cause of maxillofacial injury in the female population in countries with ageing populations, while road traffic accidents were the main cause in African and some Asian centres, especially in patients ≤65 years. Assaults remain a significant cause of trauma, primarily in patients aged 19–64 years, and they are related to alcohol use.

## 1 INTRODUCTION

The epidemiology of maxillofacial trauma varies due to socio‐economic, demographic and environmental factors, and the subject is often a young male.^1^, ^2^, ^3^ One of the main limitations of most previous epidemiological studies on maxillofacial trauma is their retrospective nature, regardless of being single‐centre, multicentric^4^, ^5^ or based on a national database.^6^, ^7^, ^8^ Although in the last 30 years women have acquired a greater socio‐economic role and consequently they have a more active participation in activities outside home, becoming more susceptible to road accidents, urban violence or other causes of injury, there is still little interest in the literature regarding the epidemiology of maxillofacial trauma in the female population, with few articles dedicated to this topic,^3^, ^5^, ^9^, ^10^ and most have focused on trauma caused by violence.^1^, ^2^, ^11^, ^12^, ^13^, ^14^, ^15^

Building upon the previous experience of the European maxillofacial trauma (EURMAT) project,^9^ the trauma team of the oral and maxillofacial surgery unit in Turin, Italy, together with other thirteen centres worldwide, launched the world oral and maxillofacial trauma (WORMAT) project. The aim of this study was to evaluate oro‐maxillofacial trauma epidemiology in the female population around the world in an attempt to provide a global picture of this phenomenon. Knowledge of these epidemiological data is critical to tailor preventive measures and to assess their proficiency, to predict trauma patterns and to effectively allocate resources.

## 2 MATERIALS AND METHODS

This study was approved by the Institutional Review Board (IRB). The authors followed all relevant guidelines of the Helsinki Declaration.

This observational prospective study collected data on female patients hospitalized for oral and maxillofacial trauma from 30 September 2019 to 4 October 2020. Fourteen maxillofacial surgery units, from five different continents, participated in this project (Table 1).

**TABLE 1:** Maxillofacial surgery units participating in the WORMAT project

Continent	Country	City	Institution
Africa	Egypt	Sohag	Maxillofacial Surgery Unit, Sohag University
	Nigeria	Ibadan	Dept. of Oral and Maxillofacial Surgery, University of Ibadan
America	Brazil	Araraquara, São Paolo Araçatuba, São Paolo	Dept. of Diagnosis and Surgery, Araraquara Dental School, São Paulo State University, UNESP, Araraquara Dept. of Diagnosis and Surgery, Division of Oral and Maxillofacial Surgery, São Paulo State University, UNESP, Araçatuba
Asia	India	Aligarh	Dept. of Oral and Maxillofacial Surgery, Aligarh Muslim University
	Iran	Mashhad	Oral and Maxillofacial Diseases Research Center, Mashhad University of Medical Sciences
	Iraq	Baghdad	Dept. of Oral and Maxillofacial Surgery, Gazi Alhariri Hospital, Medical City
	Nepal	Dharan	Dept. of Oral and Maxillofacial Surgery, B.P. Koirala Institute of Health Sciences
Europa	Austria	Salzburg	Dept. of Oral and Maxillofacial Surgery, Paracelsus Medical University
	Greece	Athens	Dept. of Oral and Maxillofacial Surgery, Hippokration General Hospital
	Italy	Turin	Division of Maxillofacial Surgery, Città della Salute e della Scienza di Torino
	Spain	Vall D'Hebron	Dept. of Oral and Maxillofacial Surgery, Hospital Universitario Vall D'Hebron
	UK	Dundee	Dept. of Oral and Maxillofacial Surgery, University of Dundee
Oceania	Australia	Sydney	Dept. of Plastic, Reconstructive and Maxillofacial Surgery, Nepean Hospital

Data were collected regarding age (0–18, 19–64, or ≥65 years), cause of trauma (fall, road traffic accident [RTA], assault, accident at work, sports injury, other), mechanism of fracture (Table 2), alcohol and drug abuse at the time of trauma, fracture site, Facial Injury Severity Scale (FISS) score,^10^ associated injuries (orthopaedic, neurological, spinal, ocular, thoracic and abdominal), day and month of trauma, time of treatment (within or after 24 h of admission), type of treatment (observational, closed reduction or open reduction with internal fixation [ORIF]) and length of hospital stay.

**TABLE 2:** Cause and mechanism of injury related to different age groups in female patients with oral and maxillofacial fractures

Type		Age group (years)			TOTAL
		0–18	19–64	≥65	
Falls (43%)					
Slipping + tripping + stumbling		13	40	85	138
Fall from height ≤3 mt		8	13	3	24
Fall from stairs		7	14	7	28
Fall from loss of consciousness		1	14	14	29
Fall from height ≥3 mt		7	14	0	21
Total		36	95	109	240
RTA (35%)					
Car without seatbelt passengers		5	47	0	52
Car with seatbelt passengers		2	10	1	13
Car with seatbelt driver		0	6	0	6
Car without seatbelt driver		0	1	0	1
Motorcycle without helmet pillion		5	28	0	33
Motorcycle without helmet driver		3	10	1	14
Motorcycle with helmet driver		1	6	0	7
Motorcycle with helmet pillion		0	4	0	4
Bicycle falls with impact on the ground		13	17	2	32
Bicycle collides against car or motorcycle		1	1	1	3
Pedestrian hit by car or motorcycle		7	19	5	31
Total		37	149	10	196
Assault (15%)					
Fist		5	45	0	50
Kick + Fist		2	10	1	13
Blunt force trauma		0	13	0	13
Kick		0	2	1	3
Firearms		0	1	0	1
Cutting instruments		0	2	0	2
Total		7	73	2	82
Sports (4%)					
Equestrian activities	Impact against opponent	1	3	0	7
	Impact against ground	1	2	0	
Team ball/ stick and racquet sports	Impact against opponent	3	0	0	5
	Impact against ground	2	0	0	
Wheeled non‐motor sports	Impact against ground	2	3	0	5
Ice or snow sports	Impact against opponent	0	1	0	4
	Impact against ground	0	2	0	
	Impact against equipment	1	0	0	
Athletic activities and individual water sports	Impact against opponent	1	0	0	4
	Impact against ground	1	0	0	
	Impact against equipment	1	1	0	
Wheeled motor sports	Impact against ground	2	0	0	2
Total		14	13	0	27
Other (2%)					
Domestic accident		0	3	1	3
Iatrogenic		1	1	0	2
Unknown		0	1	1	2
Animal attack		0	1	0	1
Accident with brother		1	0	0	1
Pathologic		0	0	1	1
Hit by friend		0	1	0	1
Hit a shop window		0	0	1	1
Total		2	7	4	13
Work (1%)					
Farm and forestry workers	Contact with a tool or machinery	0	3	0	3
Factory workers	Fall on the same level	0	1	0	1
Total		0	4	0	4
TOTAL		96	341	125	562

Abbreviation: RTA, road traffic accident.

All statistical analyses were performed using SPSS software (version 27.0; IBM Corporation). Quantitative data analysis was performed using the chi‐squared test for categorical variables and the Mann–Whitney U test or Kruskal–Wallis test for categorical and continuous variables, as appropriate. The Bonferroni correction was used to account for multiple comparisons. All statistical tests were 2‐tailed, and *p* < .05 was deemed to be statistically significant.

## 3 RESULTS

**FIGURE 1:**
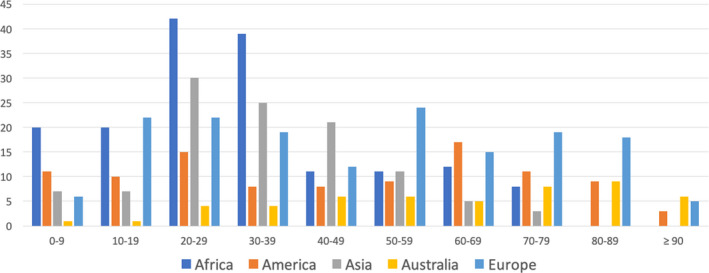
Age distribution of the female patients admitted with oral and maxillofacial trauma across the continents

During the study period, 562 of 2387 patients hospitalized with oral and maxillofacial trauma were females (24%; M:F ratio, 3.2:1) aged between 1 and 96 years (median age, 37 years; IQR—interquartile range—38). As shown in Figure 1, most fractures occurred in patients aged 20–39 years. The male:female ratio was higher in African (3.9:1) and Asian (4.0:1) units than in European, American and Australian units (mean range 2.7:1; *p* < .001 and *p* = .002, respectively; Table 3). In addition, African (median age, 19 years; IQR, 20) and Asian (median age, 32 years; IQR, 21) patients were significantly younger than European (median age, 50; IQR, 45), American (median age, 46; IQR, 46) and Australian (median age, 67; IQR, 37) patients, while the latter were significantly older than all the others (*p* < .05 for all pairwise comparisons).

**TABLE 3:** Summary of oral and maxillofacial fractures’ characteristics in the female population across the WORMAT centers

		Patients N°	Ratio M:F	Median age	Main cause of injury	Main mechanism of injury	Main site of fractures	Median (IQR) FISS	Mean (standard deviation) hospitalization stay
Africa	Egypt	140	4:1	30.50 (220)	RTA (51%)	Car without seatbelts passengers (53%)	Parasymphysis	2 (1)	2.8 (4.3)
	Nigeria	19	3.3:1	27 (32)	RTA (84%)	Motorcycle without helmet pillion (50%)	OMZc	1 (2)	4.5 (4.7)
Europe	Austria	44	2.7:1	57 (35)	Falls (52%)	Slipping + tripping + stumbling (74%)	OMZc	2 (2)	6.1 (3.8)
	Greek	19	3.2:1	53 (30)	Falls (58%)	Slipping + tripping + stumbling (100%)	OMZc	1 (0)	3.9 (4.4)
	Italy	21	3.2:1	61 (38)	Falls (71%)	Slipping + tripping + stumbling (47%)	OMZc	1 (2)	7.5 (5.9)
	Spain	33	2.6:1	15 (18)	Assault (27%)	Fist (67%)	Nose	1 (1)	2.8 (2.4)
	United Kingdom	42	2.5:1	51.50 (53)	Falls (48%)	Slipping + tripping + stumbling (85%)	OMZc	1 (0)	3.6 (6.1)
Asia	India	41	3.9:1	36 (17)	RTA (46%)	Motorcycle without helmet driver (53%)	Parasymphysis	2 (1)	3.3 (1.8)
	Iraq	7	3.6:1	29 (16)	Assault (43%)	Fist (95%)	Body	2 (1)	1.7 (0.8)
	Iran	27	3.8:1	28 (14)	RTA (74%)	Car with seatbelts passenger (35%)	OMZc/condyle	1 (1)	1.6 (0.7)
	Nepal	18	4.6:1	24.50 (43)	Falls (44%)	Slipping + tripping + stumbling (38%)	Parasymphysis	2 (2)	3.4 (3.4)
America	Brazil 1	53	2.5:1	58 (50)	Falls (46%)	Slipping (76%)	Nose	1 (2)	2.2 (1.8)
	Brazil 2	48	2.5:1	41 (44)	Falls (42%)	Slipping (70%)	Nose	1 (1)	2.3 (2.8)
Oceania	Australia	50	2.6:1	67 (37)	Falls (82%)	Slipping + tripping + stumbling (63%)	OMZc	1 (1)	2.8 (3.9)

Abbreviations: FISS, Facial Injury Severity Scale; IQR, interquartile range; OMZc, orbito‐maxillo‐zygomatic complex; RTA, road traffic accident.

The primary causes were falls (240 patients [43%]; median age, 60.5 years; IQR, 45; versus 284 patients [16%] in the male population) caused by slipping, tripping or stumbling. The incidence of falls was significantly higher in the ≥65 years group than in the other age groups (*p* < .001; Table 2). Accordingly, falls were more common in European, American and Australian units than in African and Asian units (*p* < .001). Patients experiencing falls mainly reported fractures of the middle third (43%) and lower third (43%) of the face.

Road traffic accidents were the second most common cause of injury (196 patients [35%]; median age, 29.5 years; IQR, 23; versus 870 patients [48%] in the male population) and proved to be statistically more frequent in patients ≤65 years (*p* < .001). Furthermore, RTAs were significantly more common in African and Asian centres than in the other centres (*p* < .001). In Oceania, the frequency of RTAs was the lowest (*n* = 1; 2%). Of the 72 patients who had been injured in car accidents (7 drivers and 65 passengers), 53 were not wearing seat belts, while of the 58 involved in motorcycle accidents (21 drivers and 37 pillion passengers), 47 were not wearing helmets. The remaining 66 patients were cyclists or pedestrians (Table 2). When RTAs were involved, middle third (52%) and lower third (44%) fractures of the face were common.

Assault was the third most common cause of trauma (82 patients [15%]; median age, 31.5 years; IQR, 16 versus 427 patients [23%] in the male population), 80% of whom were punched or kicked). Patients aged 19–64 years were significantly more involved in assaults (21%) than those in other age groups (7% of 0–18 years and 2% for ≥65 years; *p* < .001 for both comparisons; Table 2). The highest assault frequency was found in American units (26% of all causes), which was significantly higher than in the African and Australian units (both 8%; *p* < .001 for both comparisons), but not significantly different from the Asian and European centres (*p* = .101 and *p* = .05, respectively). When assault was the cause of trauma, middle third fractures of the face were the most frequent (60%), followed by fractures of the lower third of the face (38%).

The fourth most common cause of trauma was a sports injury (27 patients [4%]; median age, 18 years; IQR, 23; versus 128 patients [7%] in the male population). Sports injuries were statistically more common in patients aged 0–18 years (17% of all causes), rather than in older ages (*p* < .001; Table 2). European units reported the highest frequency of sports injury (10% of all causes). Injuries occurred during horse‐riding (7), team ball/stick and racquet sports (5), and wheeled non‐motor sports (5). The remaining 10 patients were injured while participating in other sports (Table 2). All patients reported fractures of the lower third (51%) or middle (49%) third of the face.

Finally, four patients (1%; median age, 40.5 years; IQR, 27; versus 89 patients [5%] in the male population) experienced maxillofacial fractures during work accidents. All patients belonged to the 19–64 year group. When trauma was related to work, fractures most commonly affected the lower third of the face (60%), followed by the middle third (40%).

Alcohol and/or drug abuse at the time of trauma was recorded in 25 patients (4%; median age, 36 years; IQR, 17). In this group, 11 had been assaulted, 9 had fallen, and 5 had been involved in an RTA. Alcohol and/or drug abuse was more common among people aged 19–64 years, but not significantly different between age groups (*p* = .053). Assaults were statistically more associated with alcohol and/or drug abuse (13% of assaulted women were under the influence of alcohol or drugs, as opposed to 3% of women with fractures from other causes; *p* < .001). On the contrary, among people who abused alcohol and/or drugs, assaults were the most common cause of trauma (44% of all causes), followed by falls.

The 526 patients in this study suffered 801 maxillofacial fractures. The middle third of the face was the site most commonly affected (443 fractures [55%]), followed by the lower third (340 [43%]) and upper third (18 [2%]). As summarized in Table 4, the orbito‐maxillo‐zygomatic complex (OMZc) was the most frequently affected site (151 fractures), followed by the nasal bones (132 fractures) and mandibular condyle (96 fractures).

**TABLE 4:** Sites and subsites of fractures in the craniofacial skeleton related to female patients' age

Site	Age group			TOTAL
	0–18	19–64	≥65	
Upper third of the face (2%)				
Anterior wall	6	9	1	16
Anterior + posterior wall	0	2	0	2
Total	6	11	1	18
Middle third of the face (55%)				
Orbito‐maxillo‐zygomatic complex	11	90	50	151
Nose	29	77	26	132
Orbital floor	5	19	18	42
Orbital medial wall	3	11	5	19
Orbital roof	4	9	4	17
Orbital lateral wall	0	5	2	7
Le Fort	1	26	7	34
Dentoalveolar	12	18	2	32
Naso‐orbital‐ethmoid complex	2	6	1	9
Total	67	261	115	443
Lower third of the face (43%)				
Condyle	25	58	13	96
Parasymphysis	18	56	8	82
Body	7	36	16	59
Angle	5	22	5	32
Symphysis	9	15	2	26
Dentoalveolar	3	18	0	21
Ramus	2	13	2	17
Coronoid	0	6	1	7
Total	69	224	47	340
TOTAL	142	496	163	801

The median FISS score was 1 (IQR 2), and patients involved in RTAs showed the highest FISS scores (median 2). Concomitant injuries were reported in 203 (36%) patients: orthopaedic (108 patients, 53%), encephalic (53, 26%), thoracic and spine (both 12 patients, 6%), abdominal (11, 5%), and ocular (7, 3%). The incidence of facial trauma did not differ by day of the week or month. The presence of at least one concomitant injury was associated with higher FISS scores (median 2, IQR 2) compared with patients with maxillofacial fractures only (median 1, IQR 1; *p* < .001).

Of all patients, 25% were treated within 24 h of trauma: 46% of those with RTA fractures, 30% of those who had experienced a fall, 14% of those who had been assaulted, 9% of those who suffered a sports injury, and 1% of those injured at work. Of all patients, 39% underwent ORIF, 36% did not receive surgical treatment, and 25% underwent closed reduction. The mean hospital stay was 3.3 days, ranging from 1.6 days in Mashhad, Iran, to 7.5 days in Turin, Italy (Table 3).

The 0–18 year age group included 96 patients (17%) with fractures mainly due to RTAs (*n* = 37) and falls (*n* = 36; Table 2). The lower third of the face was affected slightly more often than the middle third (69 and 67 fractures, respectively; Table 4). In total, 29 of 96 patients were treated within 24 h. Closed reduction was the main treatment (*n* = 42), followed by ORIF (*n* = 28) and no treatment (*n* = 26). The average hospitalization time was 2.6 days.

The 19–64 year age group included 341 patients (61%). The maxillofacial fractures in this group were mainly caused by RTAs (*n* = 149), falls (*n* = 95) and assaults (*n* = 73; Table 2). The middle third of the face, particularly the OMZc, was the most frequently affected subsite (262 fractures), followed by the lower third of the face (224 fractures; Table 4). A total of 101 of the 341 patients in this age group were treated within 24 h of trauma—156 via ORIF, 94 via closed reduction and 91 non‐surgically. The average hospitalization time was 3.5 days.

Finally, in the ≥65 year age group, there were 125 patients (22%) with maxillofacial fractures. These were mainly caused by falls (*n* = 109; Table 2). The most frequently affected site was the middle third of the face, particularly the OMZc (114 fractures), followed by the lower third of the face (47 fractures, principally in the mandibular body; Table 4). Nine of the 125 patients were operated on within 24 h. In this age group, 85 patients did not undergo surgical treatment, 35 underwent ORIF, and 5 underwent closed reduction. The average hospital stay was 3.5 days.

## 4 DISCUSSION

The most recent epidemiological reviews of maxillofacial trauma worldwide have reported that about 20% of patients are female.^12^, ^13^, ^14^ Shayyab et al.^12^ found that the male:female ratio was higher in developing than in developed countries. Boffano et al.^13^ reported lower male:female ratios in Europe, America and Australia, ranging from 1.8:1 to 6.6:1, and they were higher in Asia and Africa, ranging from 2:1 to 20:1. Both Chrcanovic^15^ and Lee^16^ observed a trend towards a reduced male bias over the last 30 years, attributed ‘to a changing workforce and to the fact that increasing numbers of women are working outdoors in more high‐risk occupations, thus becoming more exposed to RTAs and other causes of maxillofacial fractures’.^15^ In this first multicentre prospective study on this subject, the proportion of female cases of maxillofacial trauma was 24%, which is consistent with the literature.^9^, ^17^, ^18^ In addition, the male:female ratio was lower in European, American and Australian units compared with the African and Asian units.

In recent years, falls and assaults have become more frequent causes of maxillofacial trauma than RTAs in developed countries.^3^, ^13^, ^16^ Falls in the female population disproportionally affect the elderly, and the proportion of elderly persons in the general population is increasing due to their longer lifespan.^12^, ^13^, ^14^, ^15^, ^16^ It is therefore not surprising that, in four of the five European centres, and in others where the average patient age exceeded 40 years, falls (slipping, tripping or stumbling, usually followed by a ground impact) were the main cause of fractures (42%–82% of cases; Table 3). In patients aged ≥65 years, falls caused 87% of fractures and were significantly more frequent than in other age groups. Consistent with the literature, fractures of the middle third of the face (particularly the OMZc and nose) were the most common in seven of nine departments, where falls were the main cause of injury.^17^, ^18^, ^19^

The use of seatbelts in cars, wearing helmets while driving motorcycles, the strict control of speed limits and compliance with the laws related to drunk driving, combined with better road conditions and car safety features such as airbags and anti‐blocking systems, are the reasons commonly shared by several authors to explain the reduction in maxillofacial fractures caused by RTAs, especially in developed countries.^20^, ^21^, ^22^ In previous surveys in developing countries, most female oral and maxillofacial injuries were due to RTAs (53.7% in India^1^; 63.8% in Iran^11^; Table 5). RTAs were the second leading cause of female maxillofacial injury in the present study although African and two Asian centres reported rates from 46% to 84%, which were significantly higher than for the other continents. RTAs were most likely to affect those ≤65 years, which is consistent with the literature.^1^, ^2^, ^3^ Of the 130 patients involved in car or motorcycle accidents, 77% were not wearing a seat belt or helmet, and typically, female patients were passengers rather than drivers. On the whole, RTAs were associated with more complex fractures than other causes of fracture, as reflected in the FISS scores, and in centres where RTAs were the primary cause of injury, fractures of the lower third of the face were more common than those of the middle third, as reported in several previous studies.^1^, ^10^, ^12^, ^15^, ^23^

**TABLE 5:** Synopsis of the most recent literature on female oral and maxillofacial trauma

Study	Country	Study period	N°	Mean age	Causes	Site of fracture	
Roccia et al.^3^ 2010	Italy	2001–2008	365	43	Fall 43%; RTA 38.7%; assault 9.3%; sport 6.3%; other 2.7%	S	2%
						M	54%
						I	44%
Hashemi et al.^11^ 2011	Iran	2004–2006	69	33.7	RTA 63.8%; fall 19%; assault 13%; sport 2.9%; other 1.3%	S	3%
						M	28%
						I	65%
						teeth	4%
Zhou et al.^2^ 2015	China	2000–2009	250	29.9	RTA 54.8%; fall 26.8%; assault 10%; sport 2%; work 0.8%; other 5.6%	S	0.2%
						M	34.1%
						I	65.7%
Ramisetty et al.^1^ 2017	India	2005–2015	302	31.6 (median 30)	RTA 53.7%; assault 23.9%; fall 13.2%; sport 1.3%; other 6.6%	S	12%
						M	44%
						I	44%
Present study		2019–2020	562	42	Fall 43%; RTA 35%; assault 15%; sport 4%; work 1%; other 2%	S	2%
						M	55%
						I	43%

Abbreviations: I, inferior third; M, middle third; RTA, road traffic accident; S, superior third.

Assault was the third most common cause of maxillofacial fractures in the present study, and the incidence was similar to that reported by Hashemi et al.^11^ and Zhou et al.^2^ (Table 5). Consistent with the literature, these incidents more commonly involved women aged between 19 and 64 years (*p* < .001).^1^, ^3^, ^11^ As found in this study and reported in the literature, assault is a more common cause of trauma in men than in women, but nevertheless it remains a significant problem in the female population.^2^, ^9^ Intimate partner violence, in particular, frequently involves female victims and is associated with oral and maxillofacial injuries which are therefore an important marker to recognize in the emergency department setting.^24^, ^25^, ^26^ Many authors have pointed out that female patients often fail to declare the actual cause of trauma out of fear, embarrassment or low self‐esteem, so the incidence of these injuries is likely to be under‐estimated.^3^, ^23^, ^25^ In line with the literature, women were typically assaulted with fists or with a combination of fists and kicks. As also found by Gerber et al.,^23^ assault was the most common cause of alcohol and/or drug abuse‐related injuries (44% of all causes in this group). Alcohol and/or drug abuse was also significantly associated with assaults, confirming the results of other studies.^11^, ^15^, ^23^ Fractures of the middle third of the face were the main injuries. The nose, being most prominent in the face, was typically involved.^3^, ^11^, ^25^

The low incidence of maxillofacial fractures occurring during sports is also in keeping with the literature, perhaps reflecting little interest in sports among female patients, especially contact sports, and a less aggressive playing style.^27^ Although the number of injuries was relatively low, equestrian sports nevertheless caused the most fractures in this study, as also reported by several previous studies.^28^, ^29^, ^30^

Surprisingly, ORIF was performed in less than 40% of the female patients with maxillofacial fractures. These results may reflect a preference to treat nasal bone fractures and mandibular condylar fractures conservatively in adults and children, and the higher risk of surgical complications in the elderly.

## 5 CONCLUSIONS

This first prospective, multicentre epidemiological study showed that falls are the main cause of female oral and maxillofacial trauma in countries with ageing populations, particularly in the European, Australian and Brazilian centres. In contrast, RTAs were the main cause of injury in African and some Asian centres, and they were more frequent in patients ≤65 years. Assault remains a significant cause of trauma, especially in patients aged 19–64 years and with alcohol‐related injuries. Future multicentric, prospective studies are needed to monitor changes in the characteristics of maxillofacial trauma in the female population.

## CONFLICT OF INTEREST

The authors declare that there are no conflicts of interest.

## Data Availability

The data that support the findings of this study are available on request from the corresponding author. The data are not publicly available due to privacy or ethical restrictions.
